# Study protocol for a web-based personalized normative feedback alcohol intervention for young adult veterans

**DOI:** 10.1186/s13722-016-0055-8

**Published:** 2016-03-31

**Authors:** Eric R. Pedersen, Grant N. Marshall, Terry L. Schell

**Affiliations:** RAND Corporation, 1776 Main Street, PO Box 2138, Santa Monica, CA 90407-2138 USA

**Keywords:** Alcohol, Intervention, Normative feedback, Veterans, Young adults

## Abstract

**Background:**

Young adult veterans from the wars in Iraq and Afghanistan represent a population at-risk for heavy and problematic alcohol use. Unfortunately, few seek treatment for alcohol concerns and those that do seek care may drop out from lengthy multicomponent treatments. Additionally, veterans who live in rural areas and those who are not engaged in the Veterans Affairs Healthcare System are often overlooked, difficult to engage in treatment, and may not be actively seeking treatment for heavy patterns of use that may develop into an alcohol use disorder. The objective of this proposed randomized controlled trial is to develop and pilot test a brief, stand-alone Internet-based alcohol intervention with young adult veterans to help them reduce their drinking and prevent the development of problematic alcohol use.

**Methods/design:**

Recruitment and intervention is delivered entirely over the Internet to address barriers to seeking care among this at-risk group. The online intervention consists of an assessment followed by a single module of personalized normative feedback (PNF), which provides individuals with accurate information to reduce misperceptions regarding the frequency and acceptability of risky peer behavior. PNF has established efficacy as included within multicomponent interventions targeting military populations or as a stand-alone intervention with young adult college students, but has not yet been empirically supported for the at-risk veteran population. This paper describes the development of the PNF intervention content and details the protocol for the intervention study, which will utilize a sample of 600 young adult veterans to examine the efficacy of the brief PNF intervention targeted toward reducing perceived norms, intentions to drink, actual drinking behavior, and consequences. Specific subpopulations of this veteran population, including those with mental health concerns and those differentiated by level of drinking problems, reasons for drinking, and connection to peers, will be examined to support generalizability of the intervention.

**Discussion:**

This intervention has the potential to improve veteran health care by utilizing a novel approach to increase access to care, assist with drinking reductions, and prevent alcohol-related problems.

*Trial registration* ClinicalTrials.gov Identifier NCT02187887

## Background

Veterans from the conflicts in Iraq and Afghanistan [or Operation Enduring Freedom/Operation Iraqi Freedom (OEF/OIF) veterans] are a population at risk for heavy drinking and alcohol-related problems. Approximately 1 in 10 veterans from these conflicts who have sought care from the Veterans Healthcare System (VHA) between 2001 and 2010 met criteria for an alcohol use disorder (AUD) [1]. Moreover, between 22 and 40 % of these veterans drink at heavy levels that places them at risk for consequences whether or not they meet diagnostic criteria for an AUD [2–5]. Heavy use of alcohol is most prevalent among young veterans, with this group drinking at heavier rates than older veterans of the same conflicts [1]. Unfortunately, very few young veterans engaging in heavy drinking seek formal treatment to reduce use [3, 6, 7]. The Department of Defense (DoD) reports that only approximately 15 % of active duty heavy alcohol users sought treatment in the past year [8] and rates of substance use treatment among OEF/OIF service members and veterans with alcohol misuse are reported between 18 % and as low as 3 % [3, 9]. For young adult veterans, it is important to target heavy alcohol use early on so that problems do not become chronic and debilitating, and thus, more difficult to treat should the individual decide to enter treatment later on [10].

### Online interventions are a promising method to reach veteran drinkers

Heavy drinking individuals are often resistant to enter treatment due to multiple barriers such as stigma and unawareness of treatment options [11–13]. In addition to known barriers among military groups, approximately one-third of returning OEF/OIF veterans live in rural areas that may limit accessibility to hospitals and clinics within the VHA or other substance abuse treatment centers [14]. Thus, non-traditional interventions must be developed to reach young veterans with treatment needs and to help prevent the escalation of heavy drinking patterns into problematic drinking and AUDs. Online interventions represent a promising novel avenue to reach non-treatment seeking drinkers. Work with military populations suggests this group may even prefer online approaches to target alcohol use, mental health concerns, and assist with the transition back into civilian life [15–18]. Thus, online interventions represent an important avenue through which to help veterans overcome barriers to face-to-face care and receive needed services they may not have otherwise pursued.

A recent review of brief online interventions for alcohol misuse found small, yet promising, effects for reductions in alcohol use and negative consequences among adults and college students at 6 months of follow-up [19]. Although these effects are modest, online interventions represent a method of care delivery for individuals who may not have considered care for alcohol misuse otherwise, and thus represent an important area for future study. Even modest effects from a single session brief intervention may be important as an individual begins to evaluate their personal alcohol use and consider making a change, which may include enrollment in more intensive treatment. Notably, more research into stand-alone approaches that are completed solely on the Internet and that are targeted for at-risk groups of recent veterans not currently seeking care are needed. For example, only one of the included studies in the review targeted veterans [20], and those included in this “web-based” brief intervention were recruited in person from the VHA via referral or flyers posted in clinics; thus only accessing veterans already receiving services at the VHA. In addition, these participants completed an in-person assessment within the VHA and viewed personalized feedback alone in a room on an Internet-connected computer at the VHA. Therefore, there is a great need to test Internet-based interventions that use innovative online recruitment with stand-alone approaches requiring no contact with researchers or clinicians, no visits to a local VHA, and less reliance on traditional recruitment methods like flyers in a primary care clinic, which only reach those already seeking some form of care (e.g., in a primary care clinic at a VHA). These approaches would greatly expand access to care for veterans not currently enrolled in the VHA, rural veterans, and other veterans with barriers preventing them from accessing in-person services.

### Current online approaches are lengthy and have difficulty retaining participants

Despite the promising effectiveness of online interventions with veterans, most of the few existing studies are characterized by high attrition and only modest change. For example, two studies [21, 22] examined the efficacy of the multi-component, Internet-hosted drinker’s check-up [23], which is a motivational enhancement intervention with three separate modules covering assessment, presentation of normative feedback (i.e., showing how a participant drinks compared to peers), and decision-making (e.g., development of an action plan). While reductions in drinking were found at 1-month post-intervention, effect sizes were small and nearly a quarter of the participants failed to complete the three modules, which took, on average, 56 min to finish. Additionally, VHA-affiliated researchers delivered a promising online 8-week intervention to reduce drinking and alleviate symptoms of posttraumatic stress disorder (PTSD) among OEF/OIF veterans [24, 25]. Although the intervention resulted in significant reductions in both drinking behavior and consequences for intervention participants as opposed to control participants at 3-month follow-up, only half of the intervention participants completed at least four of the eight modules and only one-third of participants completed all modules. Thus, although online interventions for young veterans appear promising, designing even shorter online interventions to maximize treatment reach may be an attainable goal inasmuch as very brief interventions have been shown to yield benefits comparable to longer ones [26, 27]. Although other work has suggested that lengthier, more intensive interventions (e.g., supplementary phone calls with clinicians after completing a brief online intervention) are associated with better outcomes at 6 months and after [19], the utility of single session, stand-alone Internet programs designed to reach young veterans not currently pursuing care cannot be underestimated. That is, such approaches can reach a widespread audience, require less staffing and expertise, are accessible at all hours, and most importantly, can provide services for individuals who may have never otherwise engaged in such care. From a public health perspective, even modest effects observed may be beneficial, yet the formative work to test the feasibility of such an approach has not yet been conducted.

### Approaches solely focused on changing perceived alcohol norms are promising for young adults

One often included component of online brief interventions is the presentation of personalized normative feedback (PNF) to challenge misperceptions of peer behavior and attitudes. Indeed the inclusion of PNF is the most used technique in online interventions for adults; for example a recent review of online interventions found 16 of the 26 studies reviewed included PNF [19]. The theory and research behind PNF is that individual behavior, including drinking behavior, is influenced by perceptions regarding group behavioral or attitudinal norms [28, 29]. Given that young adults often overestimate the extent to which their peers drink or hold favorable attitudes toward alcohol consumption, misperceptions of normative behavior may be the most influential determinant of drinking behavior [30]. Thus, correcting misperceptions of peer drinking norms has become one of the prominent strategies in multicomponent interventions to prevent and reduce excessive alcohol use among the young adult population [23, 31–38], including service members and veterans [21, 22, 39].

Recent research with veterans recruited from the VHA has successfully used PNF as part of multicomponent approaches to reduce heavy drinking [39–41]. These interventions, however, rely on multiple components of behavior change, such as listing the individual’s consequences from drinking or offering information about risk factors of drinking, which greatly adds to the length of interventions. Yet, it is possible that PNF alone (i.e., normative comparison to peer drinking without any additional components) is sufficient to effect behavior change. Evaluation of several brief multicomponent interventions targeting military and non-military adults which include PNF have shown that changes in perceptions mediate the effects of these longer interventions whereas other components do not [22, 42–44]. For college students, stand-alone computer-delivered PNF has been shown to be effective at reducing drinking across eight different randomized controlled trials detailing 13 PNF-only interventions [45]. Effects for these PNF approaches are small to moderate, but underscore the promise of such a brief approach that can reach a large population of young drinkers. Despite the potential for this approach, there are no studies currently evaluating PNF-alone interventions with young adult veterans; a group at particular risk for heavy drinking and resulting problems.

There is preliminary evidence that PNF-alone approaches are appropriate for the young veteran population. For example, Williams et al. [22] found that changes in normative perceptions about the drinking behavior of other active duty service members was the only factor that mediated changes in drinking behavior over time in a multicomponent intervention. Walker et al. [44] similarly found that changes in normative perceptions after a brief motivational enhancement phone intervention mediated intervention effects on drinks per week 6 weeks later. In both of these studies, PNF was imbedded within lengthier programs and it is not known if PNF alone can effect change outside the context of these multicomponent approaches.

### The present study

The present study was designed to examine the feasibility of a stand-alone Internet-based PNF intervention for young adult veterans. We developed three aims towards this goal. The first aim was created to inform development of the single-session drinking-focused PNF intervention by collecting drinking norms in the target young adult veteran population and examining associations of different types of perceived drinking norms with alcohol use and related consequences for young adult veterans. This first phase of the study is described in detail elsewhere [46, 47]. The second aim, and the focus of this protocol, is to pilot test the developed brief PNF intervention by randomly assigning young adult veterans to either the PNF condition (N = 300) or an attention control condition (N = 300). As part of this aim, we evaluate the immediate and short-term efficacy of the intervention in changing perceived norms and reducing alcohol-related intentions, use, and consequences. Lastly, to gain a better understanding of potential effects of the intervention, we test whether reductions in perceived norms and intended drinking behaviors serve as sufficient explanatory mechanisms for any intervention effects on alcohol-related outcomes (i.e., use, consequences) and explore whether the effects of the intervention differ across meaningful subpopulations, including groups defined by demographic and military characteristics, level of drinking problems, mental health, and peer connection. In the protocol below, we describe our recruitment methods and target sample, as well as measures for outcomes, mediators, and moderators. We also discuss the format of the intervention and describe how the intervention content was informed by the first phase of the research project. The analytic plan for our study aims; that is, to examine the efficacy of the intervention and to explore mediators/moderators of intervention effects; is also described.

## Methods/design

### Participants

The Internet-based intervention is targeted toward young adult veterans. To keep with this focus, eligibility criteria include: (1) United States veteran who has been discharged or separated from the Army, Navy, Marines, or Air Force and is not currently in any of the reserve components of the armed forces, (2) between the ages of 18 and 34, (3) access to the Internet via a computer, tablet, or phone, (4) working email address, and (5) a score on the ten-item Alcohol Use Disorders Identification Test (AUDIT [48], of 4 or greater (men) or 3 or greater (women). This final criterion is based on AUDIT cutoff scores to identify those who may benefit from interventions to reduce alcohol misuse. These cutoffs with the full AUDIT measure yielded adequate sensitivity (0.87 men/0.70 women) and specificity (0.70 men/0.86 women) for hazardous drinking among veterans in prior work [49, 50]. Based on these criteria, we will recruit 600 participants who will be randomly assigned to a PNF intervention (N = 300) or an attention control condition (N = 300). Participants are anticipated to represent the demographics of the broader population of military personnel separated from the US armed forces over the past 5 years (2010–2014) for the Air Force, Army, Marine Corps, and Navy [51].

### Procedures

Participants will be recruited from the social media website, Facebook, via advertisements tailored toward “young adult veteran drinkers.” Ads target OEF/OIF veterans but veterans do not need to have been involved in these combat operations to be eligible. Facebook is becoming an increasingly viable and popular method of recruiting young adults and veterans for research and intervention purposes [25, 52–54]. During the first phase of this project, we successfully recruited 1023 validated young adult veterans using Facebook in under 1-month [47]. For the Phase 1 study, multiple validation checks were used to limit misrepresentation by participants. These included allowing only one participant per Facebook user account, ensuring consistent responses across survey items, and using screening questions to prevent and remove non-eligible individuals [55]. We will use similar procedures for recruiting participants for the intervention study. For example, participants must respond consistently to items regarding branch and rank (e.g., one could not be an “airman” in the Army; someone 34 years of age or younger would not be an “admiral” in the Navy) and will be automatically exited from the survey if inconsistent responses are provided. As we did in our previous work [47], we will also require that branch and rank are consistent with length of service, pay grade at discharge, and occupation (i.e., military occupational specialty, enlisted classification, or specialty code depending on service branch).

**Fig. 1:**
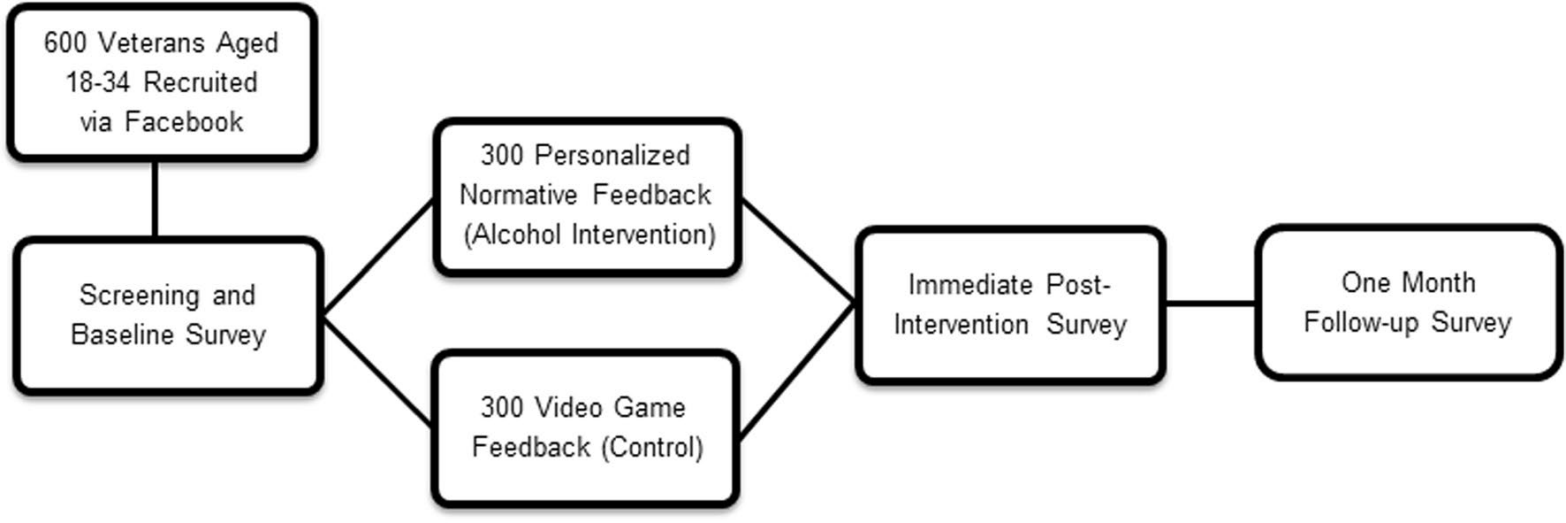
Diagram of study flow

A diagram of participant flow through the intervention study is found in Fig. 1. Participants will complete screening and baseline measures via an online survey hosted by MMIC™, a secure online data collection and management system developed by researchers at the institution where the study is based. This survey is accessible to interested participants through clicking on Facebook advertisements. After meeting eligibility criteria and completing the 15–20 min baseline survey, participants will be randomly assigned to receive the PNF intervention or an attention control condition, the latter which consists of personal video game playing feedback where one’s own video game playing behavior will be compared to perceived and actual peer norms of video game playing behavior. An attention control condition was selected over assessment-only control to limit confounding time effects inherent to the PNF intervention condition. We selected video game playing behavior for control feedback because it was not expected to associate with drinking in a manner that could confound any observed alcohol condition findings. That is, although unexpected, any potential reductions in video game playing behavior after viewing the feedback were not expected to have secondary effects on drinking reductions as something would that associated with drinking, such as if we presented feedback on sexual behavior or gambling. After reviewing their feedback (alcohol feedback for PNF participants and video game feedback for attention control participants), participants will complete a brief post-feedback survey. One month later, participants will receive, via email, a link to a 20–30 min follow-up survey.

### Development of the intervention

Phase 1 of this research project was designed to inform the content of the PNF intervention. First, since we were targeting young adult veterans in the community, we needed to document drinking norms from a community sample of young adult veterans to present during the PNF intervention. Available norms were based on active duty service members or clinical samples at the VHA which may not be generalizable to veterans in the community. Second, no previous studies had examined which reference groups should be targeted for PNF interventions with young veterans. Thus, for example, it was not clear if we should present same-gender civilian normative information or if it was necessary to present same-gender veteran information. Similarly, there was little guidance around whether veteran normative reference groups should be branch-specific, gender-specific, or both. Third, there are two types of norms typically used within PNF intervention: (1) behavioral norms, such as number of drinks per week or number of drinking days in a typical week, and (2) attitudinal norms, such as how acceptable veterans believed others found specific drinking behaviors such as drinking to get drunk or driving a car after drinking. It had not yet been determined which types of norms (behavioral and/or attitudinal) should be displayed in PNF with veterans as no study had examined the degree of association between perceived attitudinal norms and one’s own behavior in military samples. Thus, we also used the first phase of our project to examine the added utility of including attitudinal norms in addition to behavioral norms in the intervention. This research work is detailed elsewhere [46], but we describe the selection of the specific norms for the PNF based on this research below.

#### Documentation of drinking norms

During Phase 1 of the project, we collected drinking information from 1023 veterans recruited from Facebook. These veterans were demographically similar (e.g., age, gender, marital status, income, education) to young adult veterans from the American Community Survey (ACS) and to the young adult population of discharged military personnel available from the DoD. However, some differences were found. We recruited more Hispanic/Latino(a)s, fewer Black/African-Americans, more veterans of the Army and Marines, and fewer Air Force and Navy veterans than would be expected in the young adult veteran population. Thus, post-stratification weights were applied to better match our sample with the population of young adult veterans on race/ethnicity and branch of service. Details regarding recruitment of Phase 1 participants and weighted procedures are found in our other work [47]. The actual norms we will present to PNF participants are drawn from the weighted sample, and are presented in Table 1. Actual norms for video game playing are also obtained from the Phase 1 sample and will be utilized in the attention control feedback (see Table 1).

**Table 1:** Behavioral norms from the Phase 1 sample used in the intervention and control conditions

In the past 30 days…	Male veterans	Female veterans
	N = 905	N = 118
Drinking behavior		
Number of drinks per week^a^	10 drinks More than half of male veterans drink five or fewer drinks per week	9 drinks More than half of female veterans drink three or fewer drinks per week
Number of drinks per occasion^b^	3.5 drinks 63 % of male veterans drink between one and three drinks on average	3.2 drinks 73 % of female veterans drink between one and three drinks per occasion on average
Number of binge drinking days	4 days 64 % of male veterans binge drank on 3 days or fewer in the past month	3 days 69 % of female veterans binge drank on 3 days or fewer in the past month
Video game behavior		
Days played video games per week	5.1 days 31 % of male veterans play video games 3 days per week or less	4.4 days 34 % of female veterans play video games 3 days per week or less
Hours per day played video games	2.4 h 80 % of male veterans play video games between 1 and 3 h per day on average	2.1 h 80 % of female veterans play video games between 1 and 3 h per day on average
Total hours played video games per week^c^	13.5 h More than half of male veterans play video games for <10 h per week	10.2 h More than half of female veterans play video games for <7 h per week

^a^Calculated from the sum of the Daily Drinking Questionnaire (DDQ) responses

^b^Calculated from sum of DDQ divided by number of days drank in a typical week

^c^Calculated from days played video games per week × hours per day played video games

#### Selection of actual behavioral norms

We set the following criteria for determining which perceived behavioral norms would be selected for presentation in the intervention: (1) perceived norms are overestimated (i.e., drinking by veterans within a particular reference group is perceived as higher than actual), (2) perceived norms associate positively with actual behavioral drinking outcomes, and (3) actual norms are moderate enough to be meaningful for influencing behavior change. First, the mean perceived norm needed to be higher than the actual norm in the sample. For example, if veterans reported perceived drinking of peers to be seven drinks per week on average but peers actually drank an average of ten drinks per week, we would be presenting an actual norm that was higher than what most people believed the norm to be. This is contrary to the purpose of PNF interventions, which attempt to correct the *overestimation* of drinking behaviors among one’s peers. Second, perceived norms needed to associate positively and significantly with outcomes of interest (in our case: drinks per week, AUDIT scores, and binge drinking occasions) so that we can maximize the chance that changing a perception associates with reductions in those outcomes. Lastly, we wanted to select an actual norm that was moderate so we could present an actual norm that might encourage less risky drinking. That is, if we found a particular group was drinking 12 drinks per occasion on average, we would not want to present such a high norm to intervention participants. Thus, actual norms were selected for presentation in the intervention based on these criteria and using an analytic process of data we collected as part of the first phase of this project.

In the Phase 1 study [46], we considered four types of young adult referents for norms presentation: same-gender civilians, same-branch veterans, same-gender veterans, and same-branch-and-same-gender veterans. We first found that veterans in the sample reported perceptions of peer drinking behavior for all reference groups that were higher than the actual drinking of the sample. Next, we specified three outcomes of typical drinks per week in the past month, severity score on the AUDIT [48], and frequency of binge drinking occasions in the past month. Perceived civilian behavioral norms were not associated with two of our three drinking outcomes. Other work with active duty Army soldiers also confirmed that civilian norms are not associated with actual drinking behavior among those still on active duty [56]. Thus, it was determined that presentation of civilian norms would likely not be impactful on veterans if presented in the intervention.

We then found that perceived behavioral norms for same-branch, same-gender, and same-branch-and-same-gender were all consistently strongly associated with each of the three outcomes. Therefore, these were each good candidates for inclusion in the PNF. Ultimately, we selected same-gender norms based on several factors. First, research with young adults indicates that same-gender perceived norms are stronger predictors of drinking and related consequences than gender-neutral perceived norms; particularly for women [57]. Same-gender actual norms presented in PNF may also be more impactful on behavior change than gender-neutral actual norms; again, especially for women [58]. Second, the same-branch actual norms we documented in the Phase 1 sample were higher than the same-gender actual norms for several subgroups (see [46] for Phase 1 sample drinking). For example, the actual norm for drinks per week for male veterans was 10.5 and 8.7 for female veterans, but the actual norm for Army veterans was 12.1 drinks per week. Thus, the same-gender actual norms implied lower, more moderate levels of drinking compared to the same-branch actual norms, with same-gender actual norms close to levels specified as “low risk” by the National Institute on Alcohol Abuse and Alcoholism [59].

Lastly, while in some cases same-branch-and-same-gender actual norms were lower than same-gender actual norms, the Ns within some same-gender-and-same-branch groups were low. Thus, these norms were estimated with greater sampling error. The small sizes of certain subgroups could possibly raise concerns among participants in the intervention that the PNF content was based on information from too few referents to be believable. For example, we could present that a typical female Marine Corps veteran drinks about 3.4 drinks per week, but this norm would be based on just 16 female Marine Corps veterans in our sample. Thus, same-gender actual norms appeared to be the most appropriate and feasible to present during the intervention.

#### Selection of actual attitudinal norms

A goal of our Phase 1 study was also to determine whether attitudinal norms would be an appropriate component to add to the PNF. Interventions with PNF in military populations have only focused on behavioral norms [17, 22, 40, 41]. However, it is hypothesized that interventions may be more effective if they also included correction of perceived attitudinal norms since some work with college students indicates perceived attitudinal norms (e.g., acceptability of drinking behaviors such as drinking to get drunk, drinking enough to pass out) are associated with personal drinking and consequences [60–64]. Thus, similar to the behavioral norms, we set three criteria to determine whether or not to include attitudinal norms as an adjunct to the behavioral norms in the PNF: (1) perceived attitudinal norms must have an association with drinking behavior after controlling for the effect of perceived behavioral norms, (2) actual attitudes of the sample should indicate that veterans are less accepting of risky drinking behavior than they were perceived to be, and (3) actual attitudinal norms should be moderate enough to be meaningful for influencing behavior change (i.e., most veterans in the sample believe that risky drinking behaviors like “drinking to get drunk” and “drinking enough to pass out” are personally unacceptable). Attitudinal norms items are found in Table 2.

**Table 2:** Attitudinal norms by gender from the Phase 1 study

	Percentage indicating response for actual attitudes by gender					
	Males			Females		
	Never acceptable (%)	Rarely or sometimes acceptable (%)	Often or always acceptable (%)	Never acceptable (%)	Rarely or sometimes acceptable (%)	Often or always acceptable (%)
Drinking to get drunk	30	55	15	40	49	11
Males mean perception; mean actual attitudes: 3.28; 2.43						
Females mean perception; mean actual attitudes: 2.79; 2.12						
Drinking alcohol every weekend	26	49	25	32	40	28
Males mean perception; mean actual attitudes: 3.44; 2.63						
Females mean perception; mean actual attitudes: 2.56; 3.15						
Drinking to blow off steam	25	50	25	30	51	19
Males mean perception; mean actual attitudes: 3.42; 2.72						
Females mean perception; mean actual attitudes: 3.12; 2.61						
Driving a car after drinking	81	17	2	84	14	2
Males mean perception; mean actual attitudes: 2.09; 1.32						
Females mean perception; mean actual attitudes: 1.73; 1.28						
Drinking more than one drink in front of my own or others’ children	44	44	12	55	32	13
Males mean perception; mean actual attitudes: 2.74; 2.09						
Females mean perception; mean actual attitudes: 2.42; 2.00						
Drinking alcohol daily	50	41	9	55	37	8
Males mean perception; mean actual attitudes: 3.06; 1.91						
Females mean perception; mean actual attitudes: 2.81; 1.89						
Drinking alone	31	48	21	34	47	19
Males mean perception; mean actual attitudes: 3.11; 2.51						
Females mean perception; mean actual attitudes: 2.88; 2.63						
Drinking enough alcohol to pass out	67	29	4	76	23	1
Males mean perception; mean actual attitudes: 2.77; 1.57						
Females mean perception; mean actual attitudes: 2.17; 1.38						
Drinking when feeling down or depressed	44	43	11	49	40	11
Males mean perception; mean actual attitudes: 3.02; 2.10						
Females mean perception; mean actual attitudes: 2.68; 2.00						

Attitudinal norms were on a scale from 1 (never acceptable) to 7 (always acceptable)

In the Phase 1 study, we decided to be consistent across behavioral and attitudinal norms and thus looked to the effects of same-gender perceived attitudinal norms. These finding are presented in more detail elsewhere [46] and summarized here. First, we found that not all of the perceived attitudinal norms we examined were positively and significantly associated with drinking outcomes (drinks per week, AUDIT, binge drinking) when examined as zero-order correlations or after controlling for perceived behavioral norms. Only binge drinking was significantly associated with perceived attitudinal norms; however, this correlation was in a non-hypothesized direction, such that beliefs that other same-gender veterans were more accepting of risky drinking behaviors associated with *less* binge drinking in the sample.

Second, we found that, in most cases, beliefs about the attitudes of other veterans were generally reported as more permissive than the sample reported themselves. See Table 2 for means of perceived and actual attitudes by gender. For example, males perceived that other male veterans found drinking to get drunk sometimes acceptable (mean of 3.28) but reported that they found the behavior rarely acceptable for themselves (mean of 2.43). However, we found that, in general, these same-gender actual attitudes were not particularly moderate. For example, the actual attitudinal norm for drinking every weekend suggested that, depending on gender, about one-quarter to one-fifth found this often or always acceptable, while only about one-quarter to one-third found this never acceptable. This norm, as well as drinking daily (only about 50 % found it never acceptable) and drinking when feeling down or depressed (<50 % found this never acceptable; one in ten found it often or always acceptable), did not appear likely to encourage reduced drinking in the treatment sample. The two exceptions where actual attitudes seemed appropriate were driving after drinking (about 80 % found this never acceptable) and drinking enough to pass out (upwards of three quarters of veterans found this never acceptable). However, we found that belief that other veterans found drinking enough to pass out was acceptable was associated with *less* drinking behavior [46], which is counterintuitive to the theory that greater perceived attitudinal norms are associated with *more* drinking behavior.

Thus, attitudinal norms about driving after drinking appeared to be the only attitudinal norm that met our minimum inclusion criteria for the PNF intervention. However, we ultimately decided not to include this single attitudinal norm in the intervention for practical reasons. First, although the perceived norms of same-gender veterans’ acceptability of driving after drinking was positively and significantly associated with binge drinking after controlling for perceived behavioral same-gender norms, the effect was comparatively small and was non-significant for two other outcomes of drinks per week and problem drinking on the AUDIT [46]. The lack of consistent effects was particularly concerning in light of the fact that so many other candidate attitudinal norms were non-significantly associated with drinking behavior (or were significantly associated in the non-hypothesized direction) leading to concerns that this one effect was spurious. This is in contrast to the behavioral norms that were much more strongly associated with all three outcomes. Second, the current empirical support for including attitudinal norms in a PNF intervention is lacking. Including such norms without strong evidence of association with outcomes and without prior research suggesting efficacy risks undermining the intervention and could make it difficult to interpret the results. Lastly, including attitudinal norms would make the intervention harder to replicate or extend to other populations. Behavioral norms are already presented in programs with active duty and veterans [21, 22, 39, 40] and behavioral norms for presentation in PNF are already available (e.g., from DoD data, VHA outpatient data, our own community sample from Phase 1). However, attitudinal norms are not routinely collected and thus any program that includes attitudinal norms presentation would need to first document these attitudinal norms among the target reference group.

### Format of the intervention

**Fig. 2:**
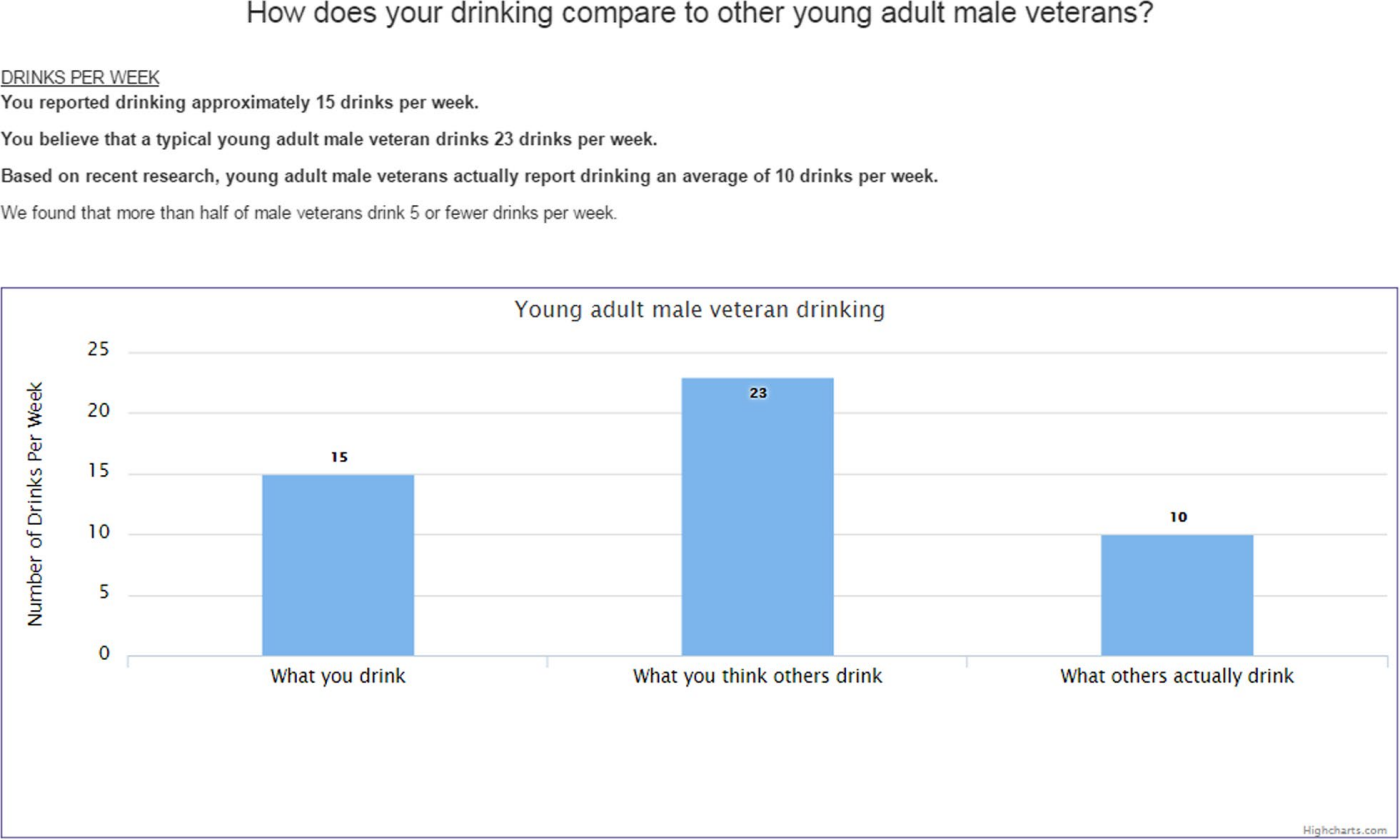
Sample of PNF for a male veteran

The format for the norms presentation, in which the behavioral norms are presented alongside information on individual use and perceived normative use [65], was informed by prior work using computer and Internet-based formats [64, 66, 67]. Based on Phase 1 work in which we found that about two-thirds of our sample completed the survey on their phones after seeing the ads on the Facebook app/Facebook website on their phones [47], we needed to make the baseline survey and intervention cleanly translatable to mobile devices. Figure 2 is an example of the PNF intervention and control conditions as expected to be viewed on laptop/desktop computers and mobile phones. In addition to viewing information on number of drinks per week in the past month, participants will also view information on number of drinks consumed per occasion and number of binge drinking days in the past month. Similar to other PNF protocols, participants will also receive information about social norms theory (i.e., why do people misperceive others’ drinking) and a description of the sample on which norms were estimated to promote credibility of the norms presented. Feedback for the attention control condition will follow the same format, with the inclusion of video game information instead of drinking (days played video games per week, hours per day played video games, total hours played video games per week).

### Analytic plan

#### Main effects of the PNF intervention

We will conduct an experimental trial of the developed PNF intervention designed to demonstrate acceptability of the intervention materials and provide preliminary data on the effects of the intervention. We will look at immediate effects of the intervention, as well as effects 1-month later. Specifically, we will determine if the PNF intervention evidences immediate and short-term changes in perceptions of peer behavior, as well as in intentions to drink alcohol (immediately) and actual alcohol use and consequences (at 1-month follow-up) relative to a control condition. We will also determine if the intervention has an effect on motivation and likelihood to reduce drinking behavior or seek further alcohol treatment to reduce drinking at both the immediate and 1-month follow-ups. We hypothesize that, compared to attention control participants, PNF participants will experience greater reductions in perceived norms and intended behavior at immediate post-intervention, as well as greater reductions in drinking behavior and related consequences when assessed 1-month post-intervention.

##### Outcome measures

Main outcomes of the intervention include changes in drinking behavior and alcohol-related consequences between intervention and control participants. Drinking in the past month will be assessed using the Daily Drinking Questionnaire (DDQ) [68], the standard measure used in norms-focused research [69–71]. Participants indicate how many drinks they consume on each night of a typical week. The DDQ allows for creation of several drinking variables including total drinks per week and average drinks per occasion, which will serve as outcomes (and are also used as part of the intervention content). We will also use a single item to assess changes in frequency of binge drinking; defined as the number of times one consumed 5/4 (men/women) or more drinks in a row during the past month. Number of alcohol consequences experienced in the past 30 days will be assessed with the 24-item Brief Young Adult Alcohol Consequences Questionnaire (B-YAACQ; [72, 73]). As this intervention is designed based on motivational enhancement approaches, we will assess if the intervention changes motivation and likelihood of reducing drinking and of seeking alcohol treatment. The latter construct will be assessed with four single-item scales rating motivation and likelihood reducing drinking/seeking alcohol treatment in the next month on a scale from 0 (not motivated/not likely) to 10 (very motivated/very likely). We will include receipt of alcohol treatment (ever, in the past 12 months) as a control variable for intention/motivation to seek alcohol treatment in the future. Intended drinking behavior in the next 30 days will also be assessed from baseline to post-intervention using a modification of the DDQ.

#### Mediator and moderator effects on the PNF intervention

We propose to investigate whether effects of the intervention on outcomes can be attributed to the proposed mediating mechanisms. We also will document intervention effects across key subpopulations.

##### Measures of mediators

Reductions in perceived norms have been found to be a major factor mediating intervention effects in interventions containing PNF alone or PNF with other components. The purpose of PNF is to change these perceptions and thus we hypothesize that PNF will reduce perceptions of peer drinking. Specifically, reductions in perceived norms at post-intervention will mediate intervention effects at 1-month follow-up such that PNF participants with greater reductions in perceived norms will benefit most from the intervention. Perceptions about alcohol use by the targeted reference group will be assessed for inclusion in the intervention content, but changes in perceptions is also identified as a mediator of intervention effects. We will assess behavioral normative perceptions with the Drinking Norms Rating Form (DNRF) [74], which is a modification of the DDQ that asks participants to consider “the drinking of a typical (gender-specific) veteran aged 18–34” when filling out the measure. This is the standard measure for assessing norms included in PNF interventions [64, 66, 67].

##### Measures of moderators

Exploratory moderators will be assessed to determine if the intervention works better for certain groups, such as less severe versus more severe drinkers, those with mental health problems (PTSD, depression) versus those without, those who drink for social reasons versus those who drink for coping reasons, and those who feel close to the PNF reference target versus those who feel distant from them. We will oversample women to obtain an N that permits us to explore whether the intervention is more/less effective for a specific gender (male/female), due to differential drinking patterns between genders in military samples [75, 76].

*Severity of drinking* We will explore whether the intervention may be appropriate for those at higher severity of drinking (i.e., possible alcohol dependence). The latter issue is important inasmuch as these individuals might arguably require further referral and more intensive counseling than could be provided in a brief intervention [48]. The 10-item AUDIT is used for screening purposes, but will also be used to assess for moderation effects of the intervention based on baseline severity of drinking. Participants with scores of eight or higher (indicative of problematic alcohol use [48] will be compared to those with scores between 4/3 (men/women) and eight. Additionally, depending on N within each score category, we may compare (a) participants with scores between 4/3 and 15 with (b) those who have scores of 16–19 and (c) those with scores between 20 and 40 to determine if this brief intervention demonstrates short-term effects for participants warranting varying levels of treatment recommendations outlined by Babor et al. [77]. We hypothesize that those with more severe drinking patterns would benefit less from a stand-alone, brief approach and would therefore warrant more intensive treatment.

*Mental health problems* Due to the higher prevalence of AUDs among OEF/OIF veterans with PTSD and/or Major Depressive Disorder [1, 78], we will investigate mental health symptoms as a moderator of intervention efficacy. In this way, we can determine if the single session intervention can be helpful in reducing drinking among those with comorbid mental health concerns. PTSD and depressive symptoms will be assessed as moderators of intervention efficacy. PTSD symptom severity will be assessed with the PTSD Checklist for DSM-V (PCL-5) [79]; a widely used measure for military and veteran populations with adequate reliability and validity for young adult military samples [80]. The PCL-5 includes 20 items related to diagnostic criteria of PTSD. Depressive symptoms will be assessed with the Patient Health Questionnaire 8-item (PHQ-8); a reliable and valid measure of depression used in research and practice for military and veterans [81]. Sensitivity and specificity are above 0.90 in military samples for the PCL-5 and the PHQ-8 [82–84]. Brief alcohol intervention studies have found that college students [85] with PTSD symptoms have reported reductions in alcohol use comparable to those without PTSD symptoms during brief interventions with counselor-delivered feedback and veterans with diagnosed PTSD reported reductions in symptoms 3-months after receiving a computer-delivered brief alcohol intervention as an adjunct to treatment as usual [20]. Other work has found that those with more severe depression and PTSD respond worse to brief alcohol interventions and substance use treatment over time compared to those without these symptoms [86–88]. As evidence is mixed and there are no studies specifically looking at PNF effects on young veterans recruited from the Internet, we will explore whether those with more severe PTSD and/or depression benefit better or worse from the intervention. Poor outcomes at 1-month for those screening for depression and/or PTSD may be suggestive of the need for more intensive dual-diagnoses treatment.

*Drinking motives* We will examine whether the intervention is appropriate for those who primarily drink for social reasons or if the intervention can also help reduce drinking for those engaging in coping-related drinking. Prior work suggests PNF may be more effective for social drinkers [67, 89]; however there is great need to examine coping drinking as a moderator of PNF intervention effects; especially since the veteran population struggles with mental health concerns that may perpetuate coping drinking. Participants will complete subscales of the Drinking Motives Questionnaire (DMQ) [90] to assess drinking for social and coping reasons. Internal reliability of the DMQ is generally >0.85 in young adult samples [91, 92]. We hypothesize the intervention, which is based on the idea that individuals are influenced to drink based on what they observe and perceive others are doing in social contexts, will work best for those who drink for social reasons.

*Closeness to reference group* Closeness to peer referents is also an important consideration as the closer one feels to their reference group, the more impactful the perceived norm of that group’s behavior and attitudes will be on behavior [70, 93, 94]. An adaptation of the Inclusion of Other in the Self Scale [95] will be included to assess how close participants feel to the referent group included in the PNF. The IOS originated as a measure of closeness to a romantic relationship partner but has been adapted to assess closeness to salient groups in other research [96, 97]. The measure contains seven pictures of two circles each; one representing the participant and the other representing the gender-specific referent group (i.e., other male [female] veterans). The two circles overlap to varying degrees and participants choose the picture that best represents how they feel toward the group. This measure has been used in other norms-focused work to examine closeness to referents [69, 70]. Since those who do not feel close to other veterans may not care how much they are drinking, we hypothesize that those who feel closer to the reference group targeted in the PNF will benefit most from the intervention.

#### Other information about measures

Measures assessing outcomes and mediators will be assessed at baseline, immediate post-intervention, and 1-month follow-up. Measures of moderators will be assessed at baseline only. Prior to baseline, participants will complete a screening survey to determine eligibility for the study. This measure will contain items needed to determine eligibility (age, branch of service, veteran status, 10-item AUDIT), demographic and military characteristic information to help describe the sample (gender, race/ethnicity, marital status, education, combat experience, zipcode to help describe rural status and proximity to nearest VHA, use of alcohol treatment services in lifetime and past 12 months), and items needed to help determine validity of the responses and help reduce misrepresentation (pay grade at discharge, occupation in the military, length of service). In addition to measures of outcomes, mediators, and moderators, participants will also complete two measures of video game playing and perceptions of the video game playing behaviors of other same gender veterans. Items assess video game playing days in the past month and typical hours per day spent playing games. This information will be used to provide PNF on video game use in the attention control condition.

### Limitations and alternative methods considered

We have considered and attempted to resolve limitations to the research plan. One limitation is use of self-report measures collected via the Internet, which could be associated with bias. However, research suggests confidential surveys enhance reliability and validity of self-report [98–101] and response rates are higher for web than mailed surveys, including among ethnic minority participants [102]. We have used similar recruitment and data collection techniques previously and have gathered representative samples of young adults in both college [103] and military populations [78]. In all recruitment materials and surveys, we ensure participants of the confidential nature of the data. Electronic methods may further provide a greater sense of anonymity, thereby reducing underreporting of undesirable or stigmatizing behaviors [104, 105]. It is also possible that for some participants the normative information presented may suggest one’s perception is lower than the actual norm. However, our process of selecting target norms is designed to minimize any iatrogenic effects. Studies with college student drinkers and abstainers have suggested that iatrogenic effects may not be detrimental during PNF interventions with young adults [106–108] and feedback can be efficacious even when presented to groups that correctly perceive they drink less than others [109, 110]. Previous research with military samples already includes PNF components with no reported iatrogenic effects [21, 22, 39]. There is also evidence to suggest that military populations do not drink as much as is traditionally perceived by the general population [75].

In addition, the use of Facebook could potentially yield a high functioning or socially-skilled sample; excluding those with mental health concerns or those isolating from peers, yet our Phase 1 study recruited from Facebook indicated this was not the case; with upwards of 50 % of the recruited sample screening for mental health concerns such as PTSD and depression [47]. Nevertheless, we have included mental health concerns, drinking motives (social vs. coping), and closeness to peers as moderators to help examine if this recruitment mechanism limits the generalizability of our study sample. Also, we elected to recruit through Facebook only and not on other websites or print media to determine the feasibility of recruitment and intervention solely through Facebook, which broadens accessibility of the approach to veterans in the community outside the VHA system. This may limit the generalizability of our sample but our prior work indicates Facebook can be used to obtain a sample similar to the broader population of young adult veterans [47]. By design, our Internet-based study excludes those without Internet access (e.g., the homeless), yet the vast majority of young adult veterans have access to the Internet [15, 16, 111].

Finally, an innovation of this approach is its brevity, yet the approach is best conceptualized as a two-session intervention which requires a degree of active participation and time. However, young people are more likely to attend to personally-relevant health information in a personalized format and the Internet is considered to be an important method of promoting access to health-related information for young adults given that young adults and military may prefer to receive health information via the Internet [15–18, 112–115]. We also use an attention control condition to help control for the time effects inherent to the intervention condition, but we do not include a no-contact control condition, which would help address the potential impact of regression to the mean and assessment effects on outcomes. Considering the scope of the project as a pilot study to determine the feasibility of a stand-alone intervention for young veterans delivered entirely over the Internet (including recruitment, assessment, intervention, and follow-up), we are not funded to assess effects after 1-month follow-up. While we include measures of intentions to reduce use and motivation to change drinking and seek further treatment, the single brief follow-up limits our ability to understand the long-term effects of the intervention.

## Discussion

This program uses a brief assessment and online intervention to target young veterans who may not otherwise seek or receive help for problematic alcohol use. The online interventions currently available to military populations are lengthy and lose many participants due to attrition. The planned research is novel in attempting to distill an online intervention into its most efficacious components to deliver a very brief intervention to a broad audience of veterans in need. This pilot intervention is the first of its kind specifically designed to target the special needs of this at-risk veteran group. The project targets areas of great importance to the field, such as developing innovative interventions indicated for at-risk groups, and the intervention is designed to be readily accessible, brief and engaging, and age-appropriate for young adult veterans as they transition from the military and readjust to civilian life. This selective prevention effort targets this specific group to examine the feasibility of an established intervention method and support the value of a full scale randomized trial effort to reduce and prevent the development of drinking problems.
